# Summary report of the Standards, Options and Recommendations for the management of patients with non-small-cell lung carcinoma (2000)

**DOI:** 10.1038/sj.bjc.6601083

**Published:** 2003-08-15

**Authors:** A Depierre, J L Lagrange, S Theobald, P Astoul, P Baldeyrou, E Bardet, B Bazelly, J M Bréchot, J L Breton, J Y Douillard, M Grivaux, P Jacoulet, A Khalil, E Lemarié, Y Martinet, G Massard, B Milleron, T Molina, D Moro-Sibilot, M Paesmans, J L Pujol, E Quoix, E Ranfaing, A Rivière, H Sancho-Garnier, P J Souquet, D Spaeth, A Stœbner-Delbarre, L Thiberville, E Touboul, F Vaylet, J M Vergnon, V Westeel

**Affiliations:** 1 Hôpital Jean Minjoz, Besançon, France; 2 Henri Mondor, Créteil, France; 3 Centre Paul Strauss, Strasbourg, France; 4 Hôpital de La Conception, Marseille, France; 5 Clinique Bizet, Paris, France; 6 Centre René Gauducheau, Saint-Herblain, France; 7 Hôpital Tenon, Paris, France; 8 Hôtel Dieu, Paris, France; 9 Centre Hospitalier, Belfort, France; 10 Centre Hospitalier Général, Meaux, France; 11 Hôpital Bretonneau, Tours, France; 12 Hôpitaux de Brabois, Vandœuvre-lès-Nancy, France; 13 Hôpital Civil, Strasbourg, France; 14 Hôpital Nord Albert Michallon, Grenoble, France; 15 Institut Jules Bordet, Bruxelles, Belgique; 16 Hôpital Arnaud de Villeneuve, Montpellier, France; 17 Centre François Baclesse, Caen, France; 18 Centre Val d'Aurelle, Montpellier, France; 19 Hôpital Lyon Sud, Pierre-Benite, France; 20 Centre Alexis Vautrin, Nancy, France; 21 Charles-Nicolle, Rouen, France; 22 Hôpital d'Instruction des Armées Percy, Clamart, France; 23 Hopital Nord, Saint-Etienne, France

**Keywords:** non-small-lung carcinoma, practice guideline

In France, primary lung cancer (all types combined) is the leading cause of cancer mortality in men, and the third in women, after breast and colorectal cancer. In 1995, lung cancer was responsible for 23.5% of cancer deaths in men and 6.4% in women. The incidence of lung cancer is higher in men than in women, and the mortality rate is nine times higher in men than in women. The lung cancer mortality rate is constantly increasing in France, particularly in the north of the country. The increase in incidence between 1975 and 1995 was more marked in women. In France, the 5-year survival rates (11.5% for men and 16% for women) are among the highest in Europe.

## OBJECTIVES

The objective of these recommendations is to define, on the basis of a critical appraisal of the best available evidence and expert agreement, clinical practice guidelines for the diagnosis and therapeutic management of patients with stage I–IV non-small-cell lung cancer. The document does not cover preoperative work-up. Management of relapsed disease will be covered in a future document.

### Methodology

The general methodology has been previously described (Fervers *et al*, 2001). For this particular Standards, Options and Recommendations (SOR), a working group of multidisciplinary experts was established by the French National Federation of Cancer Centres (Fédération Nationale de Centres de Lutte Contre le Cancer: FNCLCC) and the French Lung Society (Société de Pneumologie de Langue Française: SPLF). A literature search of *Medline*®, *Cancerlit*® and *The Cochrane Library*® up to October 1999 was performed and this was completed with references from the personal reference database of the members of the working group.

After selection and critical appraisal of this literature, the working group defined the ‘*Standards*’, ‘Options’ and ‘*Recommendations*’ (SORs) for the diagnostic and therapeutic management of patients with non-small-cell lung carcinoma based on a synthesis of the best available evidence.

‘Standards’ identify clinical situations for which there exist strong indications or contra-indications for a particular intervention and ‘Options’ identify situations for which there are several alternatives, none of which have shown clear superiority over the others (Table 1

**Table 1:** Definition of ‘Standards, Options and Recommendations’

Standards	Procedures or treatments that are considered to be of benefit, inappropriate or harmful by unanimous decision, based on the best available evidence

Options	Procedures or treatments that are considered to be of benefit, inappropriate or harmful by a majority, based on the best available evidence

Recommendations	Additional information to enable the available options to be ranked using explicit criteria (e.g., survival, toxicity) with an indication of the level of evidence

). In any SOR, there can be several ‘*Options*’ for a given clinical situation. ‘*Recommendations*’ enable the ‘*Options*’ to be weighted according to the available evidence. Several interventions can be recommended for the same clinical situation, so that clinicians can make a choice according to specific clinical parameters, for example, local circumstances, skills, equipment, resources and patient preferences. Adapting the SORs to a local situation is possible if the reason for the choice is sufficiently transparent and this is crucial for successful implementation. Inclusion of patients in clinical trials is an appropriate form of patient management in oncology and is recommended frequently within the SORs, particularly in situations where the evidence is too weak to support an intervention.

The type of evidence underlying any ‘*Standard*’, ‘*Option*’ or ‘*Recommendation*’ is indicated using a classification developed by the FNCLCC based on previously published models. The level of evidence depends not only on the type and quality of the studies reviewed, but also on the concordance of the results (Table 2

**Table 2:** Definition of level of evidence

*Level A*
There exists a high-standard meta-analysis or several high-standard randomised clinical trials that give consistent results

*Level B*
There exist good quality evidence from randomised trials (BI) or prospective or retrospective studies (B2). The results are consistent when considered together

*Level C*
The methodology of the available studies is weak or their results are not consistent when considered together

*Level D*
Either the scientific data does not exist or there is only a series of cases

*Expert agreement*
The data do not exist for the method concerned, but the experts are unanimous in their judgement

). When no clear scientific evidence exists, judgement is made according to the professional experience and consensus of the expert group (‘expert agreement’).

The document containing the SORs for the diagnostic and therapeutic management of patients with non-small-cell lung carcinoma was then peer-reviewed by independent experts. These SORs will be updated when new evidence becomes available or if there is a new consensus among the experts.

This summary report has been produced from the integral report that was validated in August 2000, and published as a monography (Depierre *et al*, 2002), a summary of version (Depierre *et al*, 2003) and on the web site of the FNCLCC (http://www.fnclcc.fr).

## IDENTIFICATION OF POSSIBLE OCCUPATIONAL RISK FACTORS FOR PRIMARY LUNG CANCER

All patients with lung cancer should have a specialist consultation to identify any possible occupational cause. If an occupational cause is identified, a declaration of occupational illness should be made (recommendation, expert agreement).

## SMOKING IN PATIENTS WITH LUNG CANCER

In patients treated for lung cancer, active smoking is an operative and postoperative risk factor and is very likely to increase the risk of a second lung, head and neck or oesophageal cancer. Patients receiving curative treatment for lung cancer should be specifically targeted with smoking cessation strategies (recommendation, level of evidence: B).

## SMOKING CESSATION STRATEGIES

### Efficacy of nicotine substitutes

In patients with a nicotine dependence, chewing gum and skin patches have been shown to be efficacious for stopping smoking (standard, level of evidence: A).

### Efficacy of behavioural treatments

Group therapy has been shown to be more efficacious than no intervention or minimal contact (standard, level of evidence: A), but it is no better than other behavioural interventions (standard, level of evidence: B1).

### Efficacy of acupuncture, hypnosis and homeopathy

There is no evidence to suggest that acupuncture, hypnosis or homeopathy are efficacious in stopping smoking (level of evidence: A).

### Efficacy of training health professionals

Training health professionals to give advice, in isolation, is probably not an efficacious strategy for smoking cessation (level of evidence: A).

## CHEMOPREVENTION OF LUNG CANCERS

No chemopreventive agent has been shown to be efficacious in the general population, in smokers, in workers exposed to occupational carcinogens or in patients who have undergone surgery for a previous tobacco-related cancer (standard, level of evidence: B1). Beta-carotene has a deleterious effect on mortality and the risk of lung cancer (level of evidence: B1). Chemopreventive agents should not be offered to patients who smoke and who have lung cancer, to patients exposed to chemical carcinogens, or to patients treated for tobacco-related cancers, who have a good prognosis (standard).

The only efficacious prevention is an anti-tobacco campaign aimed at the general population, patients exposed to carcinogens, and patients treated for a previous lung or head and neck cancer (recommendation, expert agreement).

## SCREENING FOR LUNG CANCERS

Cytological investigation of sputum has not been shown to be useful in screening for lung cancer (standard, level of evidence: B1). Regular chest X-rays can be performed in smokers and ex-smokers (option). The frequency of this surveillance, the extent of radiation exposure and the age at which screening should be initiated have not been determined (option, level of evidence: B1).

Mass screening cannot be justified, outside the setting of prospective controlled studies. These trials should assess aspects that have not been addressed previously, such as the early identification of certain markers from sputum or the usefulness of spiral CT scan in screening (recommendation).

## IMAGING IN THE DIAGNOSIS OF LUNG CANCER

### Diagnostic imaging tests

When possible, a spiral CT scan should be undertaken for diagnostic imaging as it gives the best image (option, level of evidence: D). An anterior–posterior and lateral chest X-ray, performed with a high kilovoltage technique, is essential in the diagnostic work-up (recommendation). If lung cancer is suspected, a normal chest X-ray will not be sufficient to exclude the diagnosis (recommendation). Patients with any suspicion of lung cancer, despite a normal chest X-ray, should have a thoracic CT scan (recommendation). If a suspicious lesion is observed on a normal chest X-ray, a thoracic CT scan is warranted, if treatment, other than supportive care, is envisaged; a baseline evaluation is required to assess treatment response; or a transparietal fine needle biopsy is planned (recommendation).

A CT scan image is not sufficiently specific to confirm the diagnosis (recommendation).

### Positron emission tomography (PET scanning) in diagnosis

Surgery is not recommended if the lung lesion measures less than 10 mm and if there is no fluoro-2-deoxy-D-glucose (FDG) uptake. Clinical and radiological surveillance should be undertaken for 4–6 months to ensure that the lesion does not increase in size. The upper limit of nodule size warranting this approach is not known, but it is estimated to be perhaps up to 40 mm. This policy of surveillance is particularly appropriate to minimise any unnecessary invasive active investigation of a nodule (recommendation, expert agreement).

## DIAGNOSTIC LUNG ENDOSCOPY

Sputum cytology is not specific for lung cancer, therefore histological confirmation should be obtained (standard).

### Bronchoscopy

The investigation should be performed under general anaesthetic, particularly if several samples are to be taken and the procedure is likely to be prolonged (option). Disposable bronchoscopes and biopsy clamps can be used (option). If a distal tumour is suspected, distal samples can be taken using an intensifying screen, to avoid the need to perform a transparietal fine needle biopsy (option). If the nodule is peripheral and smaller than 2 cm, the sample should be taken via a transparietal fine needle biopsy (option).

In patients with haemoptysis, emergency bronchoscopy can localise the bleeding. If there is no urgency and if abnormal lesions are visible on a chest X-ray, CT scan images can help to localise the area prior to bronchoscopy (option).

Routine haematological and biochemical tests should not replace history taking and clinical examination (recommendation). It is not necessary to perform this work-up except in high-risk patients (recommendation, expert agreement). Bronchoscopy is recommended if lung cancer is suspected and if treatment other than symptomatic treatment is envisaged (recommendation).

In patients with a proximal tumour, several samples should be taken, using different methods (recommendation, expert agreement). For patients with a distal tumour, at least one fine needle aspirate should be taken for cytology (recommendation, expert agreement).

## THORACIC TRANSPARIETAL NEEDLE BIOPSY

If the lesion is thought to be a benign tumour, a trucut biopsy needle is preferable for obtaining larger tissue samples, which will be easier to interpret (option, level of evidence: B2).

If the CT scan results are suggestive of lung cancer, and there is no other simple method for obtaining a histological diagnosis, or if the other methods have failed, a transparietal needle biopsy is recommended (recommendation).

For patients with a CT scan strongly suggestive of lung cancer in whom transparietal needle biopsy is contra-indicated, and surgery is feasible, exploratory surgery can be considered, even in the absence of histological diagnosis (recommendation, expert agreement).

## PROGNOSTIC FACTORS FOR SURVIVAL FOR PATIENTS WITH NON-SMALL-CELL LUNG CANCER

### Operable patients

**Figure 1:**
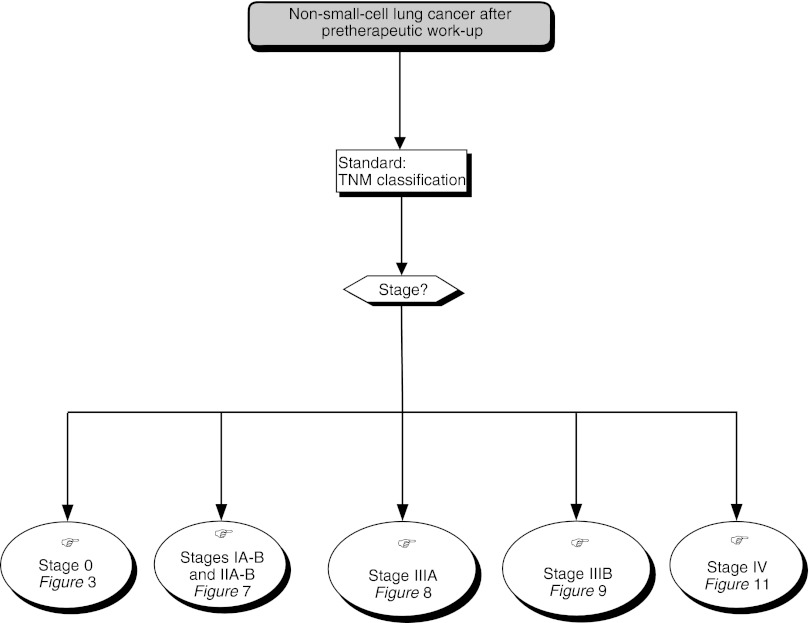
Classification by stage

Among the numerous variables studied, only the TNM (Figure 1

) staging method has been found to have a prognostic value consistently (level of evidence: B2).

### Inoperable patients

In inoperable patients, weight loss, performance status, gender, presence of metastases, high LDH concentrations, elevated white blood cell count and anaemia have been identified as prognostic factors in numerous studies (level of evidence: B2).

The value of new biological prognostic factors should be assessed in prospective studies (recommendation).

## MANAGEMENT OF PROXIMAL SUPERFICIAL LUNG CANCERS AND PRECANCEROUS LESIONS: PROBLEMS FOR DIAGNOSIS OF RADIO-OCCULT CANCERS (FIGURE 2)

### Diagnostic work-up investigations

**Figure 2:**
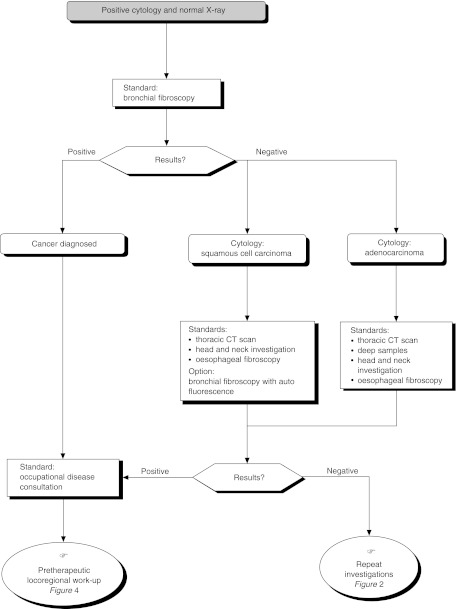
Radio-occult Lung Cancers

In patients with positive sputum cytology, bronchoscopy, CT scanning and specialised investigations to exclude a head and neck cancer should be performed (standard). In patients with negative results from initial tests of sputum cytology, bronchoscopy, with multiple samples, bronchoscopy, with autofluorescence, or regular surveillance using lung fibroscopy and CT scanning can be envisaged (option).

In high-risk patients, heavy smokers, those exposed to carcinogens or with previous lung or digestive tract cancers, bronchoscopy, with autofluorescence, can be performed (option).

### Management of carcinoma *in situ* (CIS)

Unlike other precancerous lesions, CIS should be eradicated because of the likely progression and low rate of spontaneous regression (standard, level of evidence: B2). Following treatment for a CIS, routine follow-up with bronchoscopy is indicated (standard). Local endobronchial treatment should be undertaken for patients with CIS because of its multifocal character and the frequency of secondary metachronous lesions (recommendation).

### Management of severe dysplasia

Patients with severe dysplasia should only be treated locally if the bronchial tree is involved. Bronchoscopic follow-up can be performed 2 months later (recommendation).

### Management of low-grade lesions

Low-grade lesions should not be treated and the patient should be advised to stop smoking (standard). No recommendations can be established for the follow-up of moderate dysplasia, but bronchoscopy after about 1 year is recommended (recommendation).

### Management of radio-occult cancer (Figure 3)

**Figure 3:**
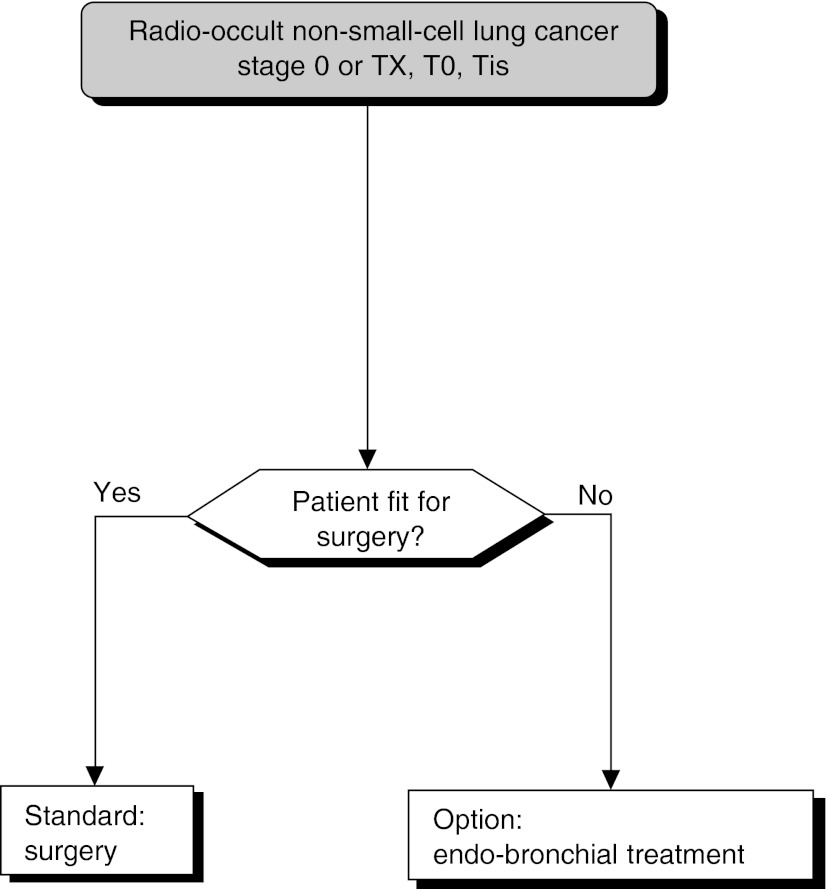
Radio-occult stage 0 or TX, T0, Tis tumours

An invasive radio-occult cancer should be treated in the same way as an invasive cancer (standard). If the CT scan shows an obstructive lesion or is suspicious for peribronchial nodal invasion, a lobectomy should be performed (standard). If a spiral CT scan does not show node invasion, local treatment (photodynamic therapy, brachytherapy or segmentectomy) is appropriate for lesions visible on bronchoscopy (option). This applies to lesions that extend less than 10 mm into the bronchial tree when they are situated in one of the segmental bronchi, and less than 7 mm when they are situated more distally. Segmentectomy with dissection of the lobar, interlobar and segmental nodes can be undertaken for lesions localised beyond the bronchoscopic field of vision or for those larger than 10 mm, if the excision margins are clear and the resected nodes are negative (option).

Patients with lesions that are visible on bronchoscopy and extend less than 10 mm into the bronchial tree (when they are situated in one of the segmental bronchi), and less than 7 mm (when they are situated more distally), should be included in randomised clinical trials assessing the benefits of new techniques. This applies as long as spiral CT scan does not show node invasion (recommendation).

## INTERVENTIONAL BRONCHOSCOPY

Endobronchial techniques are useful for symptomatic proximal obstructions (option). For patients with major extrinsic compression of the bronchial lumen, an air-tight endoluminal prosthesis can be fitted (option). Endobronchial treatment for obstructed airways is indicated for patients with symptomatic proximal obstructions prior to specific medical treatment (recommendation).

Curative bronchoscopic treatment is recommended for carcinoma *in situ*. This includes, in order of preference: cryotherapy, photochemotherapy, thermocoagulation and luminal endo-brachytherapy (recommendation).

## WHO HISTOLOGICAL CLASSIFICATION

The 1999 WHO classification for lung carcinomas, which is based on standard light microscopic criteria, should be used (standard). The diagnosis of a squamous cell carcinoma is based on the finding of intercellular junctions and keratinisation. The diagnosis of a nonbronchioalveolar adenocarcinoma is based on the finding of glandular and/or papillary structures or on the immunohistochemical detection of mucin (standard). The diagnosis of adenosquamous carcinoma is based on the finding of at least a 5% glandular component, identifiable by light microscopy (recommendation, level of evidence: D).

A carcinoma with a bronchioalveolar structure that has a fibrotic zone should be classified as a ‘classical’ adenocarcinoma (recommendation).

Immunohistochemical investigations should not be undertaken routinely for large-cell carcinoma unless they present with neuroendocrine morphology (recommendation, expert agreement). For the diagnosis of neuroendocrine tumours, the number of mitoses per mm^2^ should be stated (the area being calculated according to microscopic settings) (recommendation, expert agreement). An immunohistochemical study is recommended for fusiform-cell carcinomas with no obvious epithelial differentiation (recommendation, expert agreement).

## PROGNOSTIC VALUE OF ONCOGENES AND TUMOUR SUPPRESSOR GENES

There is no clinically useful prognostic value for the various oncogenes and tumour suppressor genes (p53, bcl-2, Ki-ras, c-erbB-2, Rb, p16) that have been identified in patients with non-small-cell lung cancer (standard).

Large prospective multicentre studies are necessary to assess the prognostic value of oncogenes and tumour suppressor genes, with the involvement of a multidisciplinary working group (recommendation).

## USE OF SERUM TUMOUR MARKERS

The use of serum tumour markers in the management of non-small-cell lung cancer is not justified (standard).

## MEDIASTINAL LYMPH NODE STAGING (FIGURE 4)

**Figure 4:**
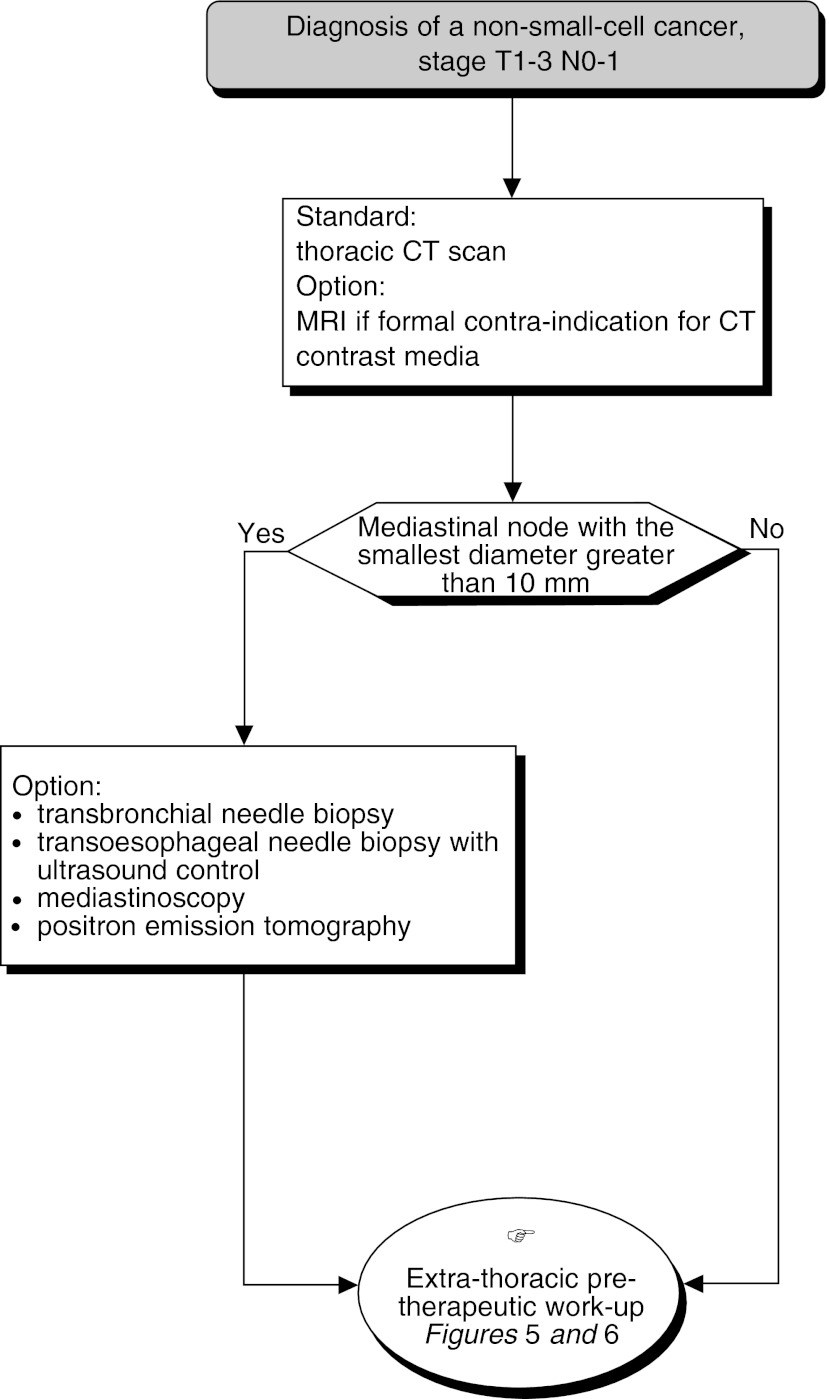
Pretherapeutic work-up for mediastinal node involvement

All patients should have at least a thoracic CT scan (standard). MRI does not give better results than CT, but it can be undertaken as an alternative in patients with a formal contra-indication to iodinated contrast media (standard).

A mediastinal lymph node is suspicious when its smallest diameter is greater than 10 mm (standard). Mediastinal lymph node samples should not be taken, outside the setting of a clinical trial, for nodes smaller than 10 mm. If there is evidence of adenopathy greater than 10 mm, and if positive results will modify the treatment plan, further investigations can be undertaken (option).

Transbronchial and ultrasound-guided trans-oesophageal percutaneous fine-needle aspiration can be undertaken in patients for whom the demonstration of lymph node invasion would signifi-cantly modify the treatment plan and therefore, their chance of survival. In these patients, mediastinoscopy should be performed, or when other techniques have given negative results (option).

The best quality result is obtained with an injection of contrast media (recommendation, expert agreement), using modern equipment (recommendation, level of evidence: A) using contiguous sections of 8 and 5 mm in the hilar region, or spiral CT machines (recommendation, expert agreement).

## INITIAL WORK-UP FOR DETECTING EXTRA-THORACIC INVOLVEMENT (FIGURE 5 AND FIGURE 6)

**Figure 5:**
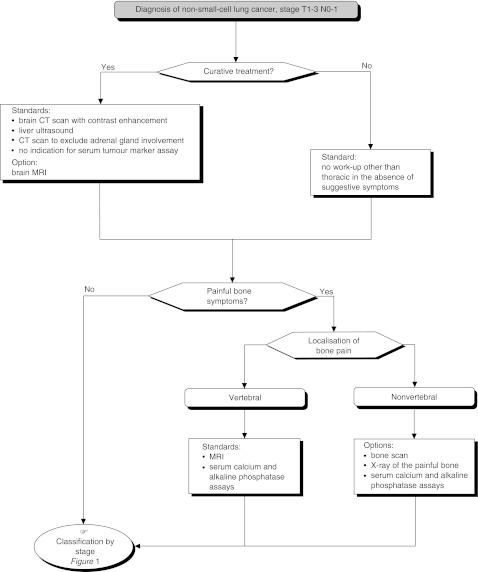
Pretherapeutic work-up for extra-thoracic involvement

**Figure 6:**
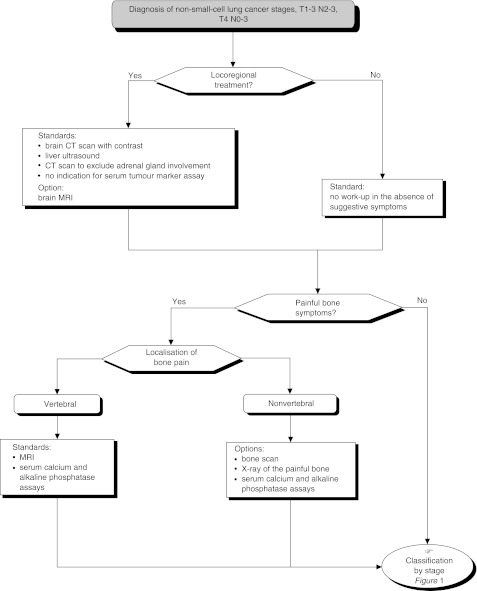
Pre-therapeutic work-up for extra-thoracic involvement

Basic work-up should be undertaken prior to any new course of therapy. The work-up should include a liver ultrasound and a thoracic CT scan that includes the adrenal glands (standard).

Patients with pain in the axial skeleton should undergo MRI as a first-line investigation. Standard radiography with CT scan or MR (with cross-sectional imaging, if necessary) should be performed in patients with bone pain elsewhere (standard). Other investigations (liver CT scan and MRI, brain MRI) should only be performed in addition to the investigations above, in certain situations (standard).

Neurological investigations can be performed depending on the stage of disease and symptoms (option). When available, brain MRI can be the first-line investigation (option). MRI is recommended for patients with a single brain metastasis (recommendation, expert agreement). Morphological detection of a single metastasis (brain, adrenal gland, etc.) requires histological confirmation, if this is the only contra-indication for chest surgery (recommendation, expert agreement).

Clinical studies should be performed to assess the impact of adrenal gland involvement, to compare brain CT scan and MRI, and to evaluate the cost-efficacy ratio for PET scanning (recommendation).

If PET scanning becomes available, it could be considered as an alternative to the current standard work-up (option).

Bone scintigraphy has limited utility because of its lack of specificity (standard).

Tumour markers have no value in this work-up (standard).

## GOOD PRACTICE GUIDELINES FOR SURGICAL TREATMENT

Surgical excision of lung cancers should be performed by an experienced chest surgeon, who operates regularly (standard, level of evidence: B2). The hospital should have the infra-structure to manage postoperative complications (standard). The decision to operate or not should be made after a multidisciplinary consultation within the institution (standard). The surgical and pathological reports should reflect the quality of the treatment and should be standardised (standard).

## GOOD PRACTICE GUIDELINES FOR RADIOTHERAPY

### Good practice guidelines for external-beam radiotherapy

Radiotherapy should conform to the guidelines of the International Commission on Radiation Units (ICRU) reports 29, 50 and 62, and the recommendations of quality assurance programmes, such as those proposed by the French Society of Oncological Radiotherapy and the French Society of Hospital Physicians (standard).

Thoracic radiotherapy should be performed using a high-energy linear photon accelerator (standard). The weekly dose, following classical application, should not exceed 10 Gy (recommendation). Split-course radiotherapy should only be performed as a palliative treatment (recommendation).

The use of recognised and validated classification systems, such as the RTOG/EORTC system, is essential for reporting treatment toxicity (recommendation).

### Good practice guidelines for endobronchial brachytherapy

The dose for endobronchial brachytherapy with a single catheter should be specified in the central plane at 1 cm from the source (standard, expert agreement). The efficacy of endobronchial brachytherapy has not yet been established.

## SURGICAL TREATMENT (FIGURE 7)

### Basis of surgical treatment

**Figure 7:**
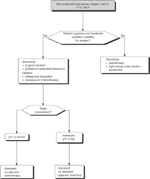
Stage I and II tumours

Surgical treatment of lung cancer should involve excision of the diseased lobe or the lung itself and ipsilateral mediastinal node dissection (standard). There is no consensus as to the extent of the node dissection.

### Operative mortality

The operative mortality rate for pneumonectomy should be less than 6%, and that for lobectomy should be less than 2% (recommendation).

### Limits for certain types of excision

Excision by lobectomy or pneumonectomy with node dissection is the basis for surgery in lung cancer (standard). Pneumonectomy carries a higher operative risk, but the results, in terms of cancer treatment, are better (standard, level of evidence: B2). Exceptionally, segmental or atypical excision can be undertaken in patients with respiratory failure or in elderly patients for whom normal excision is not possible (option).

Lobectomy with bronchoplasty is an alternative treatment to pneumonectomy in patients with node-negative, small tumours or those with respiratory failure (option). Simple surveillance is preferable to radiotherapy in patients who have undergone re-excision and who have bronchial invasion by an epithelioma *in situ* (recommendation, level of evidence: B2). Pneumonectomy, following neoadjuvant treatment, is probably associated with a higher risk of morbidity.

### Limitations related to tumour extension (Figure 8)

**Figure 8:**
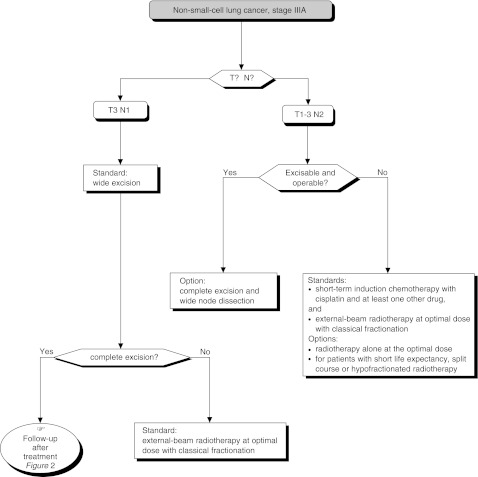
Stage IIIA tumours

In patients with stage T3 tumours, wide excision is justified for stage N0 or N1 tumours (standard). In patients with stage T1-T2N0 tumours, a single, isolated operable brain metastasis is a formal indication for surgery if the patient has no contra-indication for chest surgery (standard, level of evidence: B2).

Surgery is contra-indicated in patients with stage N3 tumours, outside the setting of a prospective clinical trial (standard).

Multiple tumour localisations should be used when undertaking curative surgery (standard). T4N0 tumours can be treated by wide excision in carefully selected patients (option). There is no consensus as to the usefulness of pneumonectomy in patients with stage N1 tumours (option). Radical excision, rather than nonsurgical treatment, is justifiable for stage N2 tumours (option).

### Surgical limitations related to patient characteristics

In strictly selected patients, age is not an absolute contra-indication for surgical excision for lung cancer (standard, level of evidence: B2). A previous malignancy outside the lung is not a contra-indication for planned curative excision (standard).

Surgery should be the treatment of choice for patients with metachronous cancers, as for patients with primary lung cancer (standard, level of evidence: D).

Cancer in a single lung after pneumonectomy can be treated by conservative resection in patients with the following criteria (option): initial cancer with a good prognosis (stage I or II), no metastases detected, no nodal involvement at mediastinoscopy, good performance status, and absence of cardiovascular disease.

In elderly patients, conservative excision techniques should be undertaken (lobectomy, segmentectomy) (recommendation). Severe vascular disease should be treated prior to lung surgery (recommendation). Lung function should be evaluated by *V*O_2_max determination. The threshold for operability is a *V*O_2_max of about 15 ml kg^−1^ min^−1^ (recommendation).

## POSTOPERATIVE TREATMENT

### Postoperative radiotherapy

Radiotherapy is not indicated in patients with stage I and II N0-N1 tumours, if excision was complete (standard, level of evidence: A). New radiotherapy techniques should be evaluated in patients with stage III tumours in the setting of a randomised clinical trial to assess their efficacy (recommendation).

### Adjuvant chemotherapy

The efficacy of adjuvant chemotherapy has not been clearly demonstrated. Adjuvant chemotherapy should only be performed in the setting of a randomised clinical trial (recommendation).

## NEOADJUVANT CHEMOTHERAPY

The need for multidisciplinary consultation is the only standard in this rapidly changing area (standard). Neoadjuvant chemotherapy can be given to patients with stage IB, II and IIIA tumours (option, level of evidence: C).

It is not known whether improving the operability of a tumour results in improved survival (option). Hence, chemotherapy or radiochemotherapy should not be routinely undertaken in patients with stage IIIA tumours, when complete excision of the tumour is uncertain (option). These patients should be included in randomised clinical trials (recommendation).

## RADIOTHERAPY AS SINGLE TREATMENT FOR STAGE I AND II TUMOURS

Curative external-beam radiotherapy, with classical fractionation, is an alternative to surgical excision only in patients with medical contraindications for surgery or for those who refuse surgery (standard, level of evidence: C).

Delivering a total dose of more than 60 Gy to the tumour mass can be advantageous if the radiotherapy technique proposed (directed or dosimetric distribution) takes into consideration the patient's respiratory function and does not increase the risk of severe radiotherapy-induced complications (standard, level of evidence: C).

Other radiotherapy techniques (modified fractionation, concomitant radiochemotherapy, high-dose rate endoluminal brachytherapy alone or in association with external-beam radiotherapy) should only be undertaken in the setting of a randomised clinical trial for patients with stage IB or II tumours (recommendation, level of evidence: D).

External-beam radiotherapy to the primary tumour volume alone, not including the mediastinum, can be considered in patients with peripheral stage IA tumours. This treatment should only be undertaken in patients with proximal stage IA and stage IB tumours in the setting of a randomised controlled trial, comparing localised irradiation to the tumour volume with a large field, encompassing tumour and mediastinum (recommendation, level of evidence: D).

The volume treated should include all macroscopic tumour and a safety margin of 1.5–2 cm (recommendation, level of evidence: D).

## TREATMENT OF LOCALLY ADVANCED CANCERS

### External-beam radiotherapy

When treating locally advanced non-small-cell lung cancers, external-beam radiotherapy should be of optimal quality and should deliver a minimum dose of 60 Gy with classical fractionation (standard, level of evidence: B1).

For patients with a reduced life expectancy, this radiotherapy can be delivered as a split course or in a hypofractionated mode (option).

Hyperfractionated and accelerated hyperfractionated radiotherapy should only be performed in the setting of randomised clinical trials (option).

The efficacy of conformal radiotherapy in the treatment of locally advanced non-small-cell lung cancer should be assessed in randomised clinical trials (recommendation).

### Radiosensitisation

The combination of weekly chemotherapy (platin-based and others) with radiotherapy should only be performed in the setting of a randomised clinical trial (recommendation).

### Induction chemotherapy

Patients with locally advanced tumours and a performance status of 0 or 1 should receive a combination of short sequential induction chemotherapy, containing at least cisplatin and one other drug, and conventional radiotherapy (standard, level of evidence: A).

### Concomitant radiotherapy and chemotherapy

It is possible to deliver chemotherapy and radiotherapy concurrently (recommendation). Treatment schemas should be evaluated in the setting of a randomised clinical trial (recommendation).

### Surgery

Stage N3 tumours remain a contraindication for classical surgery (standard). There is no justification for combining radiochemotherapy (adjuvant or neoadjuvant) with surgery in patients with stage N3 tumours outside the setting of a randomised clinical trial (standard).

Surgical excision remains justified in patients with stage N2 cancer because of the good quality of local control obtained (option). Patients with stage T4-N0 cancer can undergo curative surgical excision (option).

### Management of locally advanced non-small-cell lung cancers (Figure 9)

**Figure 9:**
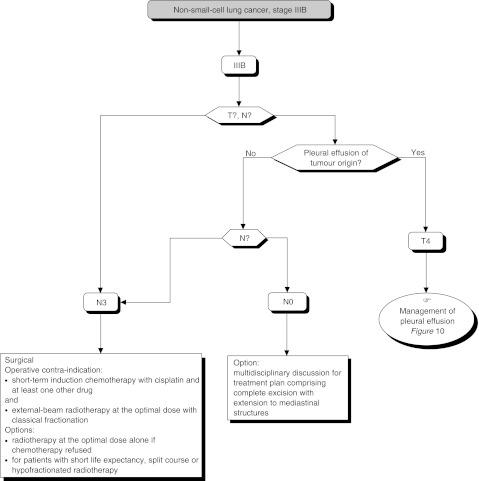
Stage IIIB

The treatment of choice is radiotherapy aimed at local control and/or improved survival (standard). Surgical excision can be undertaken in carefully selected patients, particularly those with a T4N0 tumour (option).

External-beam radiotherapy should be of optimal quality (high-energy 9 MV photons, with individualised lead shielding and computerised dosimetry) (standard). A total minimum dose of 60 Gy, with classical fractionation, should be delivered to achieve local control (standard). Wide surgical excision, associated with perioperative radiotherapy, gives a survival rate of between 20 and 35% in carefully selected patients with T4N0 tumours, although the operative mortality is about 10% (standard). Essential surgical staging, using mediastinoscopy, should provide guidance for the therapeutic decision-making process (standard).

Radiotherapy, at a dose of 60 Gy can be delivered as a split course with a rest period of 2–4 weeks (option). Hypofractionated radiotherapy gives a better quality of life for patients with less than 3 months life expectancy (option). Hyperfractionated radiotherapy does not seem to be more efficacious and has higher toxicity. Accelerated radiotherapy is an alternative to conventional radiotherapy for improving local control, but results in greater toxicity thus excluding combination with chemotherapy (option).

The efficacy of conformal radiotherapy in the treatment of locally advanced non-small-cell lung cancer should be assessed in a randomised clinical trial (option). The combination of a weekly injection of cisplatin with split course radiotherapy can improve local control and even improve survival, but results in significant gastrointestinal toxicity (option). The sequential combination of induction chemotherapy (containing cisplatin and at least one other drug) with conventional radiotherapy can reduce the rate of metastases and improve short-term survival, particularly in patients with a good prognosis (option). Induction chemotherapy alone or in combination with moderate dose radiotherapy (40–45 Gy), prior to complex surgical excision, may be indicated in selected patients with stage IIIA or IIIB cancers (i.e., those with potentially excisable tumours, and a good performance status) (option). Induction treatment can increase the risk of acute respiratory distress syndrome (ARDS) after pneumonectomy. These complex and toxic treatments should be undertaken, whenever possible, in the setting of a randomised clinical trial (option).

## MANAGEMENT OF PLEURAL EFFUSION (FIGURES 9 AND 10)

### Diagnosis of pleural effusion

**Figure 10:**
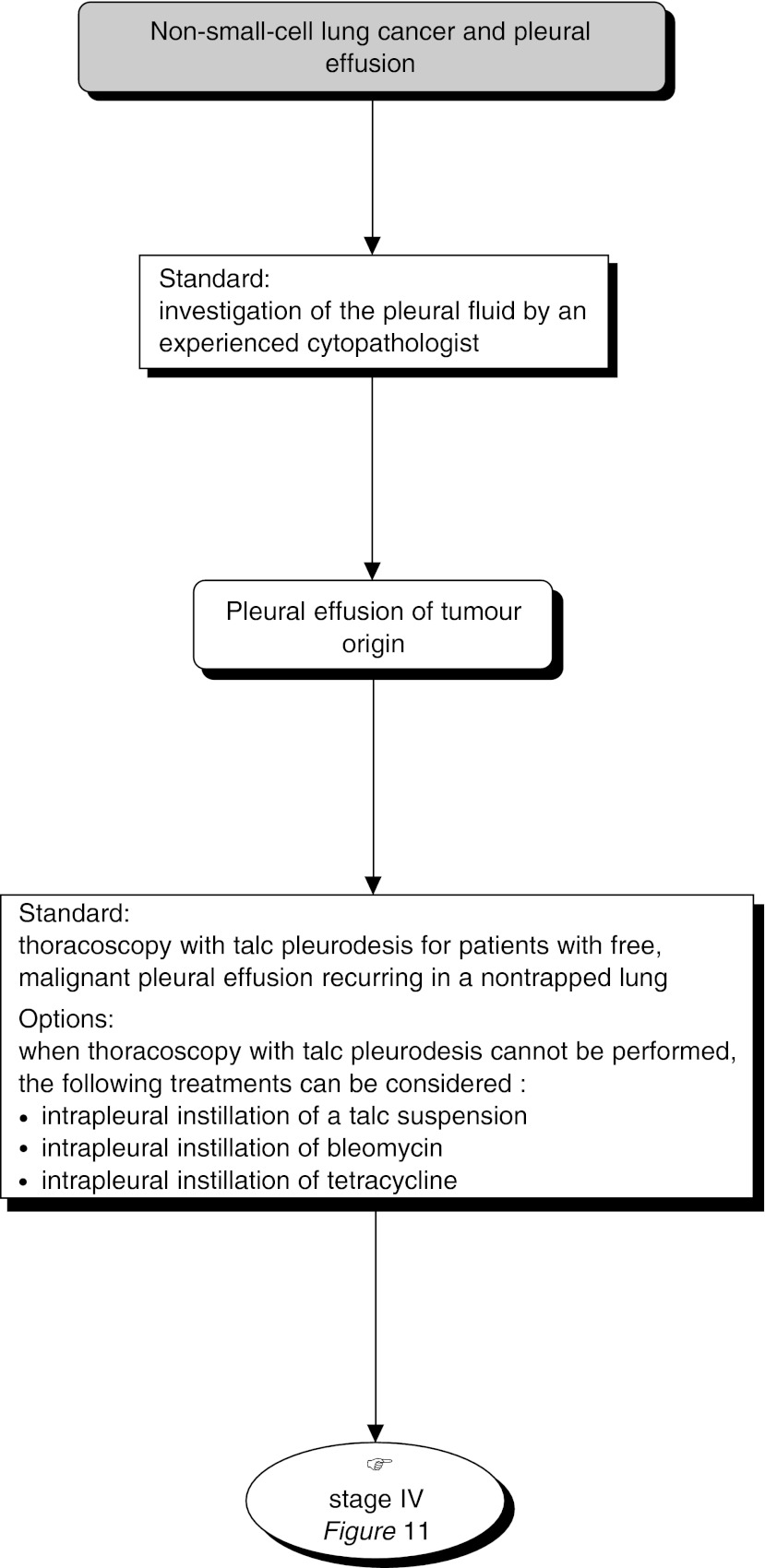
Management of pleural effusion with tumour origin

In patients with localised tumours, pleural effusion should be investigated (via cytology or histological sampling) to determine whether it is neoplastic or benign in origin (standard). The investigation should be performed by an experienced cytopathologist (standard). If locoregional treatment is not indicated, it is not necessary to perform thoracoscopy (recommendation, expert agreement).

### Treatment of pleural effusion

Thoracoscopy with talc pleurodesis is the standard treatment for patients with malignant pleural effusion recurring in a nontrapped lung (standard, level of evidence: B1). When thoracoscopy with talc pleurodesis cannot be performed, the following treatments can be considered (options):

- intrapleural instillation of a talc suspension;
- intrapleural instillation of bleomycin;
- intrapleural instillation of tetracyclines.

## TREATMENT OF METASTATIC CANCERS (FIGURE 11)

### Single metastasis

**Figure 11:**
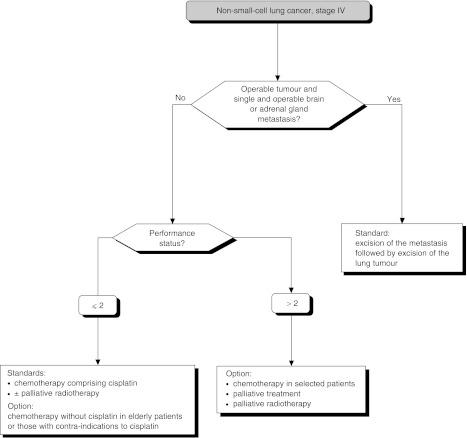
Stage IV tumours

Excision of the primary tumour and the metastasis can be undertaken in patients with operable non-small-cell lung cancers and a single brain metastasis (standard, level of evidence: C) or adrenal gland metastasis (standard, level of evidence: D).

### Role of chemotherapy

Chemotherapy containing cisplatin should be offered to patients with stage IV non-small-cell lung cancer and a performance status of 0 or 1 (standard, level of evidence: A).

### Chemotherapy in elderly patients

Chemotherapy without cisplatin can be considered in elderly patients (option, level of evidence: C).

### Treatment for multiple brain metastases

The standard treatment for patients with multiple brain metastases is 10–12 sessions of radiotherapy at a dose of 3 Gy each, given in five sessions per week (standard, level of evidence: C).

Chemotherapy can also be considered (option, level of evidence: C). Randomised clinical trials should be undertaken to assess which drugs are the most efficacious and to confirm results already obtained (recommendation). Survival without CNS recurrence should be evaluated. The evaluation of radiotherapy and its role in relation to chemotherapy (concomitant or sequential) should be investigated (recommendation).

## EVALUATION OF TREATMENT RESPONSE

### Investigations to be performed to evaluate the treatment response

A thoracic CT scan should be performed before and after treatment to evaluate the treatment response (standard, level of evidence: B2).

### Re-evaluation after treatment

In patients with a complete or partial response, as shown by CT scan, the role of fibroscopy to formally assess the tumour status should be evaluated in the setting of a clinical trial (recommendation, expert agreement).

### Evaluation of extra-thoracic lesions

Liver and adrenal gland metastases should be formally assessed prior to chemotherapy (standard). Bone metastases and pleural effusions should not be used for treatment response evaluation (standard). Skin metastases can be used to assess response (option).

### Principles of the treatment response evaluation

The following principles are recommended (expert agreement):

- record and classify all lesions present at the beginning of treatment;
- define which lesions will be used to measure response, giving preference to lesions that can be easily measured on a CT scan
- follow the WHO rules for evaluating global response;
- verify that the initial sample sites are histologically negative to confirm a complete response;
- fibroscopy is not warranted for re-evaluation in patients with lesions that are stable or are progressing as shown by CT scan;
- take into consideration the quality of the response in terms of the duration of response and especially, improvement in symptoms and quality of life.

## FOLLOW-UP AFTER TREATMENT (FIGURE 12)

**Figure 12:**
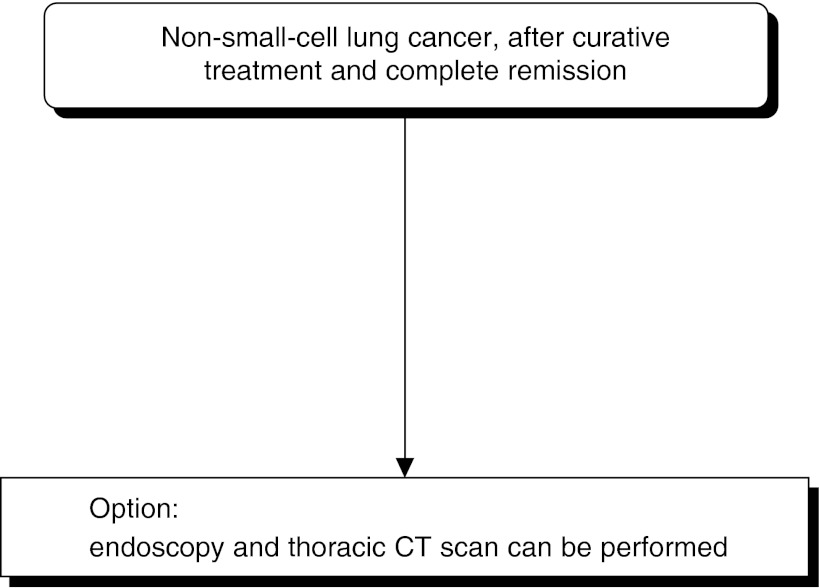
Surveillance after treatment

Follow-up should be performed with fibroscopy and CT scans (option, expert agreement). Multicentre randomised clinical trials should be undertaken to compare a defined follow-up strategy with no follow-up and with different follow-up strategies. These trials should evaluate the patients' quality of life and the cost effectiveness of the strategy (recommendation).

## Appendix

### Reviewers

JP Basuyau (Centre Henri Becquerel, Rouen), P Bonnette (Hôpital Foch, Suresnes), J Cadranel (Hôpital Tenon, Paris), MF Carette (Hôpital Tenon, Paris), JC Carrier (Membre réseau ONCORA, Centre hospitalier, Villefranche-sur-Sâone), JP Chevalier (Membre réseau ONCORA, Centre Hospitalier, Annecy), C Clary-Meinesz (Hôpital Pasteur, Nice), B Coudert (Centre G François Leclerc, Dijon), F Dhermain (Centre Henri Becquerel, Rouen), B Dubray (Centre Henri Becquerel, Rouen), I Durand-Zaleski (Hôpital Henri Mondor, Créteil), P Durieux (Hôpital Georges Pompidou, Paris), N Eche (Institut Claudius Régaud, Toulouse), M Frenay (Centre Antoine Lacassagne, Nice), B Gasser (Hôpital civil, Strasbourg), I Gormand (Membre réseau ONCORA, Infirmerie Protestante, Lyon), P Grenier (Hôpital de la Pitiè-Salpétrière, Paris), P Icard (CHU Côte de Marne, Caen), A Joveniaux (Centre Oscar Lambret, Lille), L Kaluzinski (Centre Hospitalier Louis Pasteur, Cherbourg), G Kantor (Institut Bergonié, Bordeaux), JP Kleisbauer (Hôpital Sainte Marguerite, Marseille), T Le Chevalier (Institut Gustave Roussy, Villejuif), C Le Pechoux (Institut Gustave Roussy, Villejuif), C Lévy (Centre Antoine Baclesse, Caen), F Lesaunier (Centre Antoine Baclesse, Caen), J Maublant (Centre Jean-Perrin, Clermont-Ferrand), P Mere (Membre réseau ONCORA, Centre Léon-Bérard, Lyon), JC Normand (Centre Hospitalier Lyon Sud, Pierre-Bénite), B Padovani (Hôpital Pasteur, Nice), M Pérol (Hôpital de la Croix Rousse, Lyon), T Philip (Centre Léon Bérard, Lyon), JP Pignon (Institut Gustave Roussy, Villejuif), A Prevost (Institut Jean Godinot, Reims), P Ravaud (Hôpital Bichat, Paris), I Ray-Coquard (Centre Léon Bérard, Lyon), P Rebattu (Centre Léon Bérard, Lyon), M Riquet (Hôpital Laënnec, Paris), P Thomas (Hôpital Sainte-Marguerite, Marseille), V Trillet Lenoir (Centre Hospitalier Lyon Sud, Pierre Bénite), C Tuchais (Centre Paul Papin, Angers), M Untereiner (Clinique Claude Bernard, Metz), T Urban (CHU, Angers), F Varlet (Cabinet médical, Bethune), P Verelle (Centre Jean Perrin, Clermont-Ferrand), A Vernenègre (Hôpital du Cluzeau, Limoges) and A Voloch (Centre médical Parot, Lyon).
